# Journal impact factors and the future of open access publishing

**DOI:** 10.1002/acm2.14083

**Published:** 2023-06-26

**Authors:** Susan L. Richardson, Samantha G. Hedrick

**Affiliations:** ^1^ Swedish Cancer Institute Seattle Washington USA; ^2^ Thompson Proton Center Knoxville Tennessee USA

1

In the history of academic publishing, there may not have been a more drastic philosophical and foundational change than the one from conventional printed communications to online dissemination. With this change in platform comes a host of economic and ethical transformations. This development leads those involved in publishing to re‐evaluate the meaning of journal impact factors (JIF), the value of open access (OA), and the budgetary consequences of these changes. We must also consider how we can still incorporate equity, diversity, and inclusion (EDI) into our scientific publishing process. This editorial will discuss some of the history of these topics and describe the implications for JACMP.

## 2 JOURNAL IMPACT FACTORS

In 1955, Eugene Garfield published a paper in *Science* in which he proposed a methodology for tracking and assessing scientific publications.^1^ The idea was to let scientists evaluate how their work was received and allow historians to assess the importance of these articles.^2^ With time, it evolved into collating the number of citations per journal, which eventually became the modern journal impact factor. Today, *Nature*, the *New England Journal of Medicine*, and *Cancer* are publications with some of the highest JIFs depending on the year. Journals routinely display their high impact factors proudly, hoping to invite submissions based on this number alone, implying enhanced benefit and reward to authors. Criticisms of JIF are multiple: it can encourage self‐citation, it can be skewed by a highly cited article,^3^ it is calculated by data not readily available to the public and has been described as “crude and misleading.”^4^ Interestingly, all of these assessments were made by the editors of, or editorials published in *Nature*. Even retracted articles can be cited and contribute to JIF. From an EDI perspective, articles that have been circulated in English or written by an author with an English‐sounding name receive more citations.^5^ Additionally, only journals that are listed in the commercial Thomson Scientific database (now Clarivate^TM^) are included, which are predominantly English language journals published in the United States.^6^ Furthermore, names from unfamiliar languages often have typographical errors in citations, introducing geographical bias.^7^ This is a world‐wide phenomenon as found by Nielsen et al, in which they reported the global citation inequality index is on the rise, particularly in the medical sciences.^8^ If *Nature* critiques its own self‐assessment of value using JIF and JIF embraces known, scientifically proven biases, should not the community at large not heed the cry for alternatives?

If the JIF needs replacement, what are other alternatives? One is the *h‐index*, which was developed to quantify an *individual's* research output, rather than a journal's.^9^ This methodology has been adopted by ResearchGate and Google Scholar and is prominently displayed next to the author's profile, as well as Elsevier. The h‐index also has valid criticisms and may not correlate with scientific reputation.^10^ Another interesting indicator is the *Altmetric*, which includes the media's response to publications, incorporating *social* media mentions including Twitter, Facebook, and Reddit. Indeed, a recent publication of the author's own work in February of 2023 in JACMP was tweeted and had over 6000 views at the time of this writing, which is quite impressive considering her meager following. Wiley reports the full text views at the time of this writing was 1800 with an Altmetric score of 10 (very high for a recent publication). Currently, the number of citations of the work is 0, exemplifying that citations in the modern era are no longer an accurate measure of article viewing or use and do not take into account timing and article publication cycles. Moving away from JIF and towards other metrics allows authors to self‐promote and provide access to their work without self‐citation to the benefit of all. That said, most journals are still using the JIF (including the JACMP) and it is not disappearing any time soon. Furthermore, many researchers are attracted to so‐called “mega journals,” which are characterized by being OA, high publication fees, and distribute more than 2000 articles in a given year.^11^ For comparison, in 2019, JACMP published 264.

The Declaration on Research Assessment (DORA) was constructed in 2012 to address the concerns and offer alternatives on JIF. It calls for the elimination of journal‐based metric evaluation (including JIF) for scientist funding, appointments, and promotions.^12^ It suggests publication entities encourage responsible authorship practices (such as requiring specific contributions of each author), create article‐specific assessment metrics rather than journal‐centric ones, and adopt policies that allow full and complete references in articles. Wiley, the world's largest publisher and the publisher of JACMP, is one such company that has signed onto the DORA agreement. Unfortunately, the adoption of DORA recommendations has been slow within academic facilities.^13^

## 3 OPEN ACCESS

While earlier examples existed, the boom of open access (OA) publication models started around the year 2003. The Berlin declaration^14^ stated that “the internet fundamentally changed the practical and economic realities of distributing scientific knowledge.” Anticipating this change ahead of time, JACMP started as an online only journal in 2000 and could possibly be the first OA medical journal.^15^ Online publications were fully cemented in 2013 when the Obama administration mandated that US federally funded research be freely available to the public within one year of publication.^16^ Open Access, similar to JIF, has its advantages and disadvantages.

The most often touted benefit of OA publications is access to “all”—anyone with an internet connection can access the millions of publications on the web (and at the JACMP) without cost. Unfortunately, almost 3 billion people in the world (∼37%) have never used the internet.^17^ Even so, OA models allow authors to bridge gaps and allow scientific discourse. There are many different types of OA models. In a *gold* model, the authors will pay a fee to make their work available to an OA journal, called an Article Processing Charge (APC). This is the fee model adopted by the JACMP. A *hybrid* model allows the author to pay extra to have their article OA in a conventional subscription journal.

Another advantage of OA is notoriety and publicity. According to a 2021 report from Wiley, OA articles were downloaded three times as much as subscription articles. Hybrid OA articles were downloaded four times as many. Both methods resulted in twice the citation rate compared to subscription articles and OAs received 4.5 times the Altmetric score.^18^

While reading an OA article is free, publishing in this model is not. Academic publishing can be a business of high profits—the industry generates over 20 billion dollars per year. Elsevier has a profit margin approaching 40%.^19^ In order to sell their product (a publication platform), they must offer high quality services, often described in terms of rapid turnaround times and their impact factor (and so we have come full circle). In a critical view of OA, the European Federation of Academies of Science and Humanities issued a statement which decried OA as a “hollow promise” due to APCs.^20^ This proclamation exemplifies that OA and APCs benefit those researchers in large academic institutions likely to have agreements with publishers, while ignoring the needs of the Global South, smaller institutions, or industrial fields. According to Wikipedia, the average per‐journal APC is $1600 USD. In March 2022, the average monthly pay in Africa was $758 USD.^21^ The JACMP charges $730 USD, with waivers and discounts available to first time authors, students, residents, or authors from certain countries.^22^ For comparison, *Nature* charges $11690 for Gold OA. There are alternatives outside of the Thomson Scientific (Clarviate^TM^) community, however, that serve the Global South, including the previous host of JACMP, the Public Knowledge Project or PKP.^23^

Furthermore, while not an issue for reputable publishers, there exists a pervasive issue of predatory journals vying for OA APC fees. Not only is the author done a financial disservice, but predatory journals lack quality control and cause harm by disseminating misinformation. In a stunning sting operation, John Bohannon submitted a fake article to 304 separate OA journals.^24^ By his own admission, the paper's experiments were “hopelessly flawed” and results were “meaningless.” More than half (157) of the journals accepted his bogus paper. About 60% received a final decision without peer review. Publishing in predatory journals not only allows a “pay‐to‐pad” model for one's CV, but also creates the problem of non‐experts unable to differentiate between respectable research and junk science.^25^ Particularly important in medical fields, publishing in predatory journals can have negative consequences on patients and outcomes.^26^

## 4 THE FUTURE OF PUBLISHING

With the current issues and challenges faced by academic publishing, what will the next decade look like? A few relevant topics are discussed below.

- Reimagining peer review

Peer review is currently thought of as one of the most important components of high‐quality scientific publication.^27^ However, there is an inadequacy of competent, expedient reviewers,^28^ and most peer review is uncompensated by the journals that request it. That is, the reviewers must freely volunteer to read and comment on the article to contribute to the good of science at large and the well‐being of the publication journal. Peer review is also extremely inefficient; an enormous amount of time (15 million hours) is spent re‐reviewing papers that have been previously rejected each year.^29^ Evidence is lacking that peer review substantially improves scientific papers^30^ so what should we conclude about some so‐called predatory journals that don't have peer review? Burdening academics with reviewing colleague's work only takes away time from their own publications. It can also be slow and frustrating on the part of the authors. Reviewers must also be incentivized in an appropriate way^31^; ideas that are circulating include journal discounts on APCs or contributions to research departments.^32^ Peer review could also be acknowledged as part of the academic workload. Peer review has also been shown to be extremely biased when not double masked (as it is with the JACMP), but the masking process also removes information about the diversity of the authors and reviewers. As described in the first part of this editorial, journals must start to understand who is involved in each step of the publication process.^33^

- The adoption of Preprints

Preprints are a free method of sharing research articles prior to publication without peer review. Each submission receives a Digital Object Identifier (DOI), which makes them instantly citable and provides a permanent link on the internet. Authors can make their work public and receive comments or feedback on their article. Rapid turnaround on articles (typically less than 24 h) can allow dissemination of information in a time of need. For example, the most highly read article on preprints.org at the time of this writing is on adoption of cloth masks during the COVID‐19 pandemic.^34^ With the surge of post‐pandemic preprinting, journals are beginning to allow and even encourage its use. As articles with pre‐prints have been found to have more citations and online mentions, journals should anticipate their increased adoption.^35^ The anticipated support of the preprint methodology may offer unseen challenges to authors and journals including the theft of ideas, challenges of copyright, and an overall flavor of lack of scientific integrity.^36^ Wiley has currently adopted the policy of allowing preprints with a suggestion to update the preprint with a link to the final article after publication.^37^ Authors should be aware that the use of preprints removes the masking one direction, and should consider this prior to submission.

- The use of Artificial Intelligence (AI) and ChatGPT

Automated tools may help journals streamline their review and publication processes. As described by Schulz et al., automated pre‐screening may help with improved reporting prior to review, or enhancing the peer review process.^30^ They suggest that statistical errors, ethical lapses (such as plagiarism), and citations of retracted works are all easily identified with one such automated tool. This could reduce the workload on reviewers and improve article quality such as flagging those with incorrect formats. On the downside, the use of AI has been found to include and replicate bias.^38^ The study by Checco et al. found that due to the required historical training method of AI, that cultural and organizational biases may be “frozen into the code.”

The use of automated query and response tools such as ChatGPT^39^ is also on the rise. Kappel took this question directly to the source and asked ChatGPT how it might affect scientific publishing.^40^ ChatGPT indicated that it could contribute in four ways: Improving the accuracy and quality of the writing, allowing faster review and turnaround times, providing more accessible and personalized writing, and creating new forms of scientific writing and research. While many of these items are a welcomed improvement to the publication workflow, the ethics, policies governing, and overall acceptance of automated methods into the journal publishing process have yet to be established.

There are many challenges faced by publishers and scientific journals by the wide‐spread use of the internet and the development of open access. It is not a perfect system, and many criticisms are valid. Reviews take a long time and are subject to bias. Reviewers are unrewarded for their efforts. Journal impact factors are becoming archaic, but no metric is perfect. New tools are being developed but editors are not yet sure how to incorporate them into the process. All of these challenges will be faced by the JACMP and other open access journals. On an aspirational note, in 2018, the European Commission and European Research Council launched “cOAlition S,” an initiative (Plan S) that supports worldwide open access for research funded by public grants.^41^ Among others, the World Health Organization and the Bill and Melinda Gates foundation are funders of Plan S. If enough entities agree that this is the correct path forward, we may see all journal platforms becoming open access and solve some of the financial problems therein.
